# Supplementary figures and images for: JNK Signalling Regulates Self-Renewal of Proliferative Urine-Derived Renal Progenitor Cells via Inhibition of Ferroptosis
Source: Cells. 2023 Sep 2;12(17):2197. doi: 10.3390/cells12172197 (PMC10486975; doi:10.3390/cells12172197)

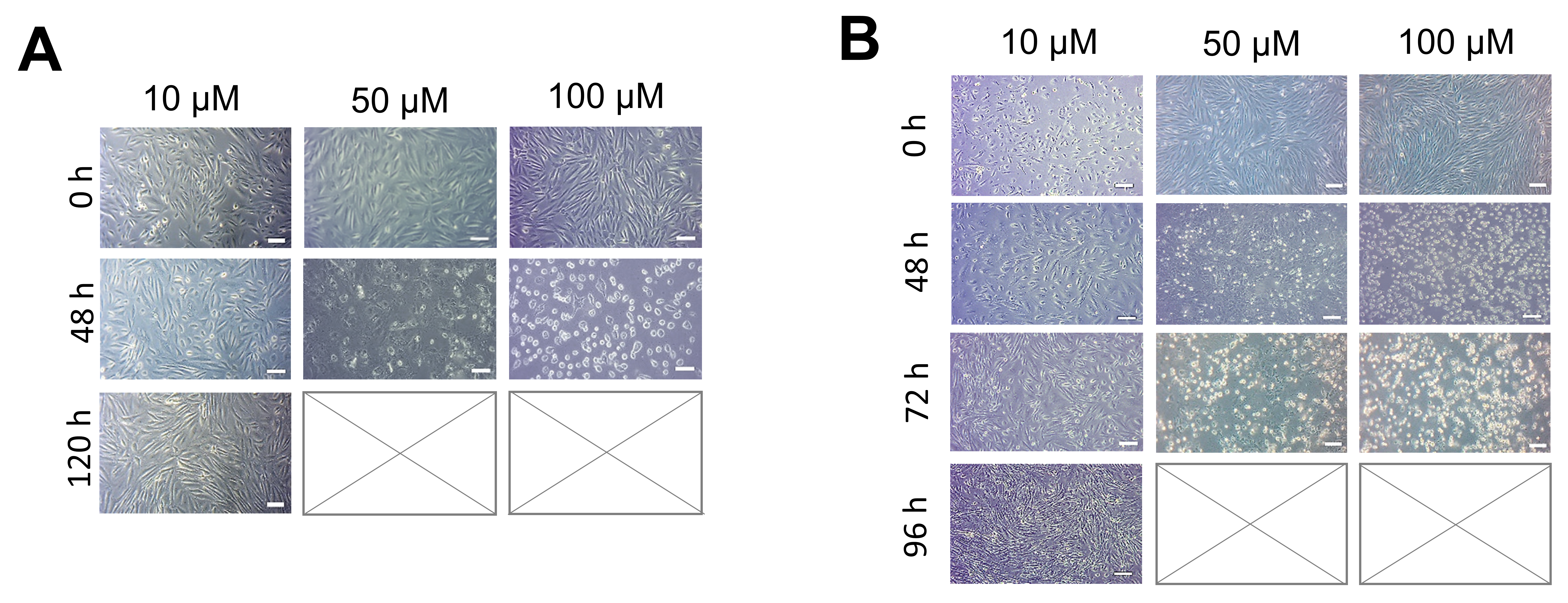

Supplement: Supplementary file 1 [file cells-12-02197-s001.zip › Figure S1.tif]

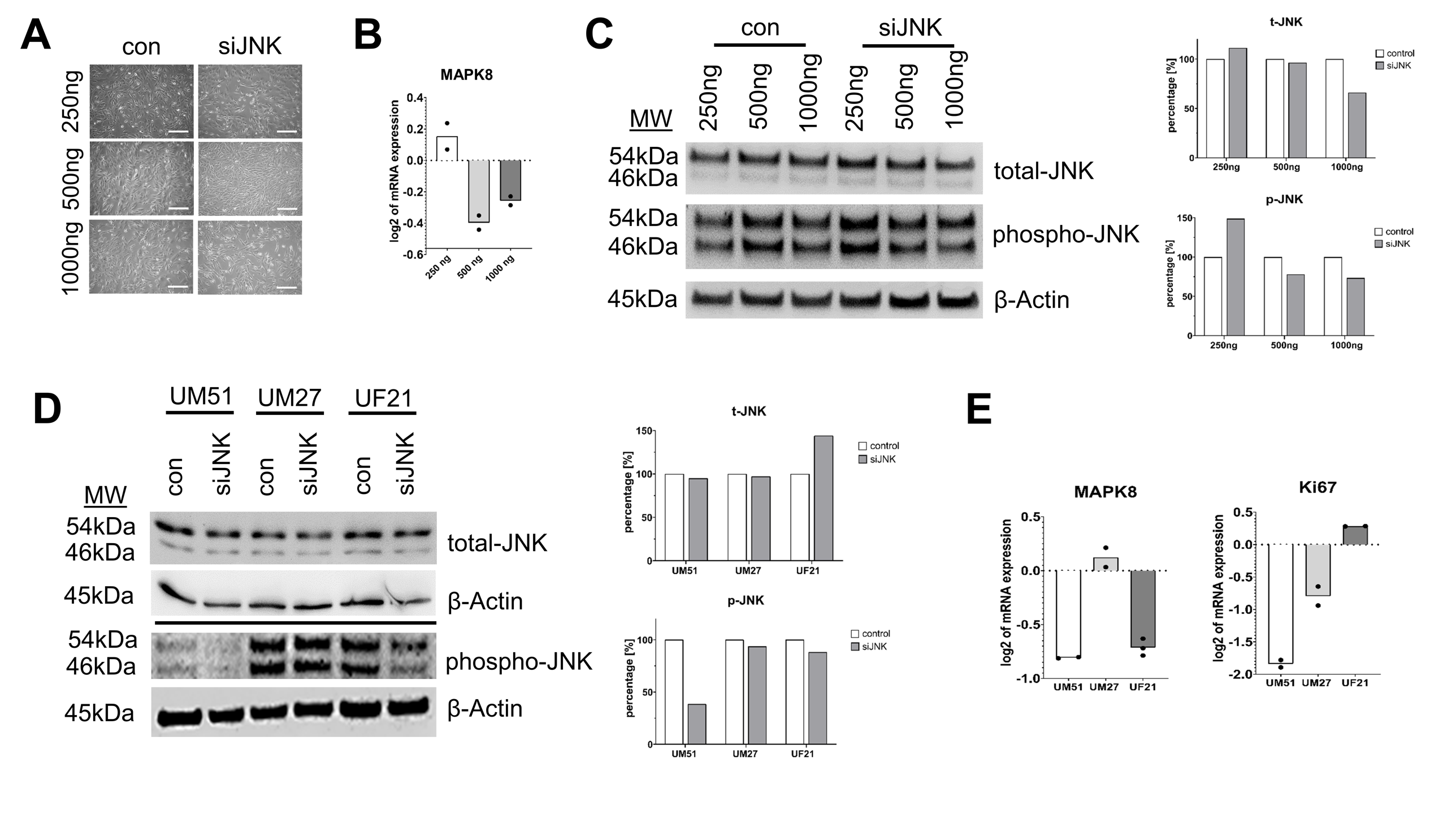

Supplement: Supplementary file 1 [file cells-12-02197-s001.zip › Figure S2.tif]

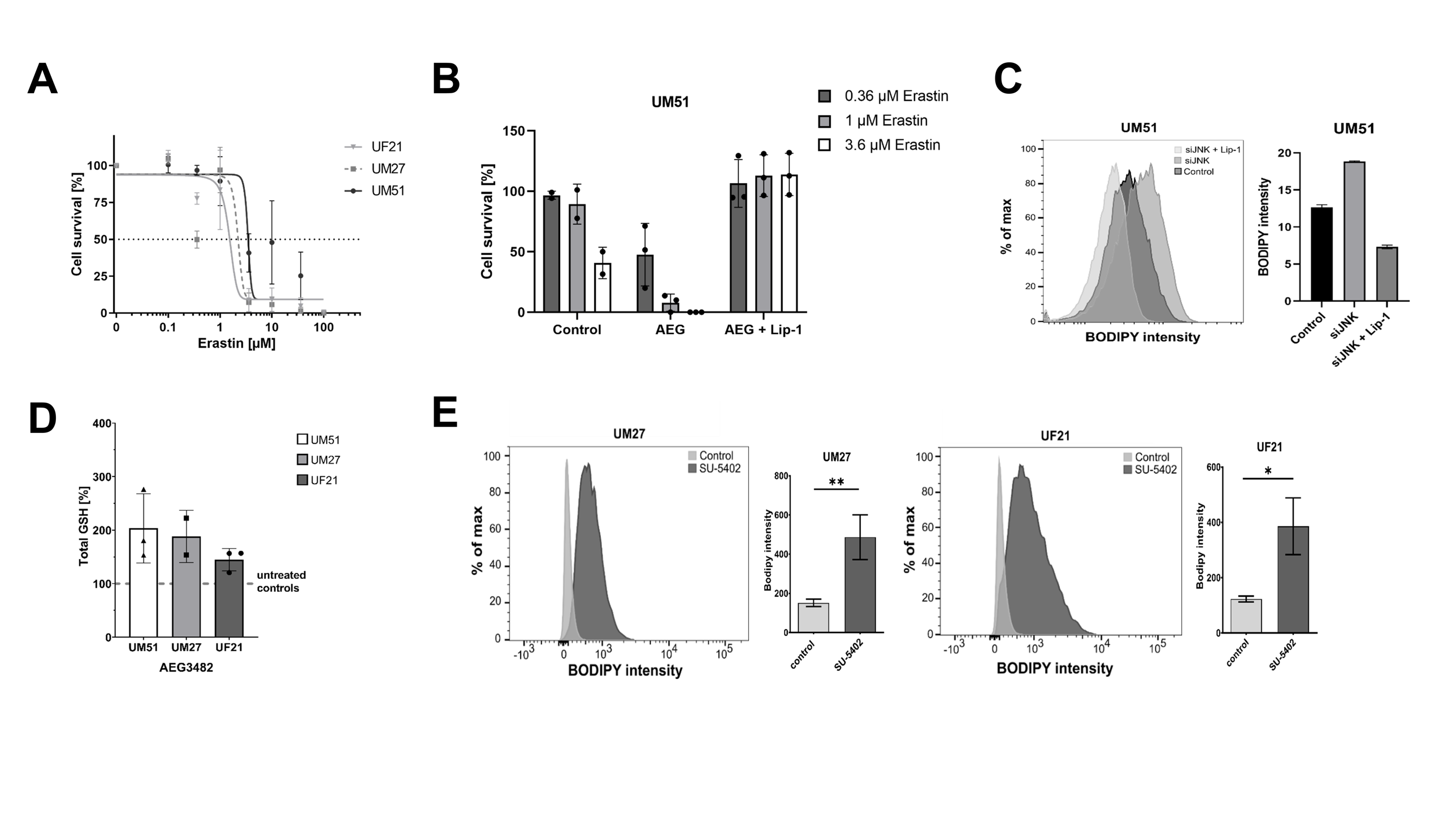

Supplement: Supplementary file 1 [file cells-12-02197-s001.zip › Figure S3.tif]
